# A prospective, randomized, double-blind, placebo-controlled study on efficacy and safety of Ashwagandha root extract (*Withania somnifera*) for managing menopausal symptoms in women

**DOI:** 10.3389/frph.2025.1647721

**Published:** 2026-01-05

**Authors:** Isukapalli Vani, Gudla Muralidhar, Bade Srinivas Rao

**Affiliations:** 1 Department of Gynaecology, Government Medical College & Government General Hospital (Old RIMSGGH), Srikakulam, Andhra Pradesh, India; 2 Department of Clinical Research, Government Medical College & Government General Hospital (Old RIMSGGH), Srikakulam, Andhra Pradesh, India

**Keywords:** Ashwagandha root extract, menopause, safety, efficacy, randomized controlled trial

## Abstract

**Background:**

Menopausal hormone therapy (MHT) is one of the most recent therapeutic options available for the management of menopausal symptoms. MHT is used in healthy symptomatic women under 60 or within 10 years of menopause without contraindications. Still, as many menopausal women use both MHT and antidepressants, safer alternatives are needed. Herbal remedies like Ashwagandha can offer a safer alternative to existing therapies. Ashwagandha aids in hormonal balance, vitality, and reduces stress and fatigue.

**Objective:**

The study aimed to assess the efficacy and safety of Ashwagandha root extract (ARE) for managing menopausal symptoms.

**Methods:**

A randomized, double-blind, placebo-controlled study included 60 women aged 45–55 years who received either ARE or a placebo (PL) for 56 days. The primary outcome was a change in the Menopause Rating Scale (MRS) score from baseline to 56 days. Secondary outcomes were changes in serum hormonal parameters [estradiol, progesterone, luteinizing hormone (LH), and follicle-stimulating hormone (FSH)], hot flash events, Short Form-12 Health Survey (SF-12) (Quality of Life) score, and Perceived Stress Scale-10 (PSS-10) from baseline to day 56. Tolerability was measured using Patient Global Assessment of Tolerability to Therapy. Safety outcomes, such as change in severity and frequency of adverse events, were also assessed from baseline to day 56.

**Results:**

At the end of the study, the total MRS score reduced significantly (*p* < 0.0001) with ARE intervention, in psychological (*p* < 0.0001), somatic (*p* < 0.0001), and urogenital (*p* < 0.0001) domains as compared to the PL group. Similarly, ARE group showed improved serum estradiol (*p* < 0.001) and progesterone (*p* < 0.001) levels, and increase in SF-12 scores (*p* < 0.001), while presenting reduced serum FSH (*p* < 0.001) and LH (*p* < 0.001), hot flashes events (*p* < 0.001) and PSS-10 scores (*p* < 0.001) compared to PL.

**Conclusion:**

Ashwagandha root extract can be a potential herbal intervention for managing menopausal symptoms in healthy women.

**Clinical Trial Registration:**

https://ctri.nic.in/Clinicaltrials/pmaindet2.php?EncHid=OTk3Mw==&Enc=&userName=, CTRI/2022/02/040551.

## 1 Introduction

Menopause is defined as the stoppage of menstrual periods for 12 consecutive months, signaling the end of ovarian function and permanent amenorrhea. A gradual shift from active to inactive ovarian function occurs over several years. Besides its association with aging, menopause involves significant biological and psychological changes in women (1, 2).

The reduction in estrogen and progesterone release during menopause makes women more susceptible to psychosomatic issues. The predominant symptoms are hot flashes, sweating, and libido alterations. However, other specific concerns associated with menopause are vasomotor symptoms, sleep disruptions, urogenital issues, breast and joint pain, cognitive changes, and mood disorders such as depression and anxiety (3, 4).

The primary treatment for menopausal symptoms involves a combination of menopausal hormone therapy (MHT), pharmaceutical antidepressants, and lifestyle changes. However, MHT is linked to a higher risk of venous thromboembolism, stroke, cardiovascular disease, gallstones, and breast cancer. In addition, women frequently discontinue antidepressants due to their significant side effects. It has been suggested that MHT is not effective in addressing psychological manifestations (1, 5–7). So nowadays, women are moving towards alternative options such as herbal remedies. One such herb used traditionally is Ashwagandha.

Ashwagandha, scientifically referred to as *Withania somnifera*, belonging to the family Solanaceae, has been utilized in Ayurveda for centuries. Ashwagandha has been utilized for millennia as a Rasayana due to its multitude of health benefits. It is a potent adaptogen that enhances the body's resilience to stress, supports cell-mediated immunity, and possesses antioxidant capabilities (8). It is a renowned herb that is highly valued for its ability to balance, energize, rejuvenate, and revitalize the body system (9–11).

Many clinical investigations have shown that Ashwagandha root extract (ARE) has a variety of physiological functions, including anti-inflammatory, neuroprotective, adaptogenic, and cognitive-enhancing properties (12). It has been utilized as an alternative treatment for hormonal disorders, in particular for infertility and sexual dysfunction. ARE has shown benefits in improving sexual desire and sexual dysfunction. A study conducted by Dongre et al. reported that ARE enhanced sexual function, sexual arousal, lubrication and lowered sexual distress in healthy women (13). It has also been shown to relieve mild to moderate climacteric symptoms during perimenopause in women. A study by Gopal et al. demonstrated that Ashwagandha significantly reduces the total MRS score and significantly increases in serum estradiol and reduces follicle stimulating hormone (FSH) and luteinizing hormone (LH) concentrations (10).

Despite this, the mechanism of ARE influence on the reproductive system is not fully understood; it might be related to its adaptogenic, anti-inflammatory, and antioxidant effects. Another possible mechanism could be Ashwagandha's GABA mimetic action, stimulating gonadotropin-releasing hormone and thus improving the hormonal balance (12).

Given these potential effects of ARE supplementation on reproductive health in women, the present study evaluated the efficacy and safety of ARE managing menopausal symptoms in women.

## 2 Materials & methods

### 2.1 Study design

This was a 56-day, prospective, randomized, double-blind, placebo-controlled, parallel clinical trial designed to evaluate the efficacy and safety of ARE in women with menopausal symptoms. The trial was conducted at Govt. Medical College & Govt. General Hospital (Old RIMSGGH), Srikakulam, Andhra Pradesh, India, between February 28, 2022, and November 30, 2022. Written informed consent was obtained from all participants in their preferred language before enrollment. A comprehensive explanation of the study objectives and expected outcomes was provided to each participant before obtaining consent. The enrollment of participants commenced on February 28, 2022. The study consisted of two site visits (baseline visit—day 1, end of study—day 56), and one telephonic follow-up visit (day 28). All assessments were carried out at the study center during visit 1 (day 1) and visit 3 (day 56), while a telephonic follow-up visit was conducted to verify drug and protocol compliance and collect details on any adverse events (AEs) without any formal assessment. The 56 days (8 weeks) were deemed to capture meaningful changes in key biochemical and psychological endpoints while ensuring optimal participant compliance and safety (10, 11).

### 2.2 Ethical approvals

The clinical study protocol was approved by the Institutional Ethics Committee (IEC), Govt. Medical College & Govt. General Hospital (Old RIMSGGH). The trial was prospectively registered with the Clinical Trials Registry of India (CTRI) with registration number CTRI/2022/02/040551. The study was conducted as per the ethical principles outlined in the Declaration of Helsinki (2013 revision), Good Clinical Practice (GCP) guidelines, and the Consolidated Standards of Reporting Trials (CONSORT) statement.

### 2.3 Study population

#### Inclusion criteria

Healthy women aged 45–55 years with a clinical diagnosis of menopause with no history of hormone therapy or antidepressant treatment in the past 3 months were included in the study. Demographic parameters included a body mass index (BMI) within the range of 20–30 Kg/m^2^ and a history of 10 or more hot flash events per week. Participants who were willing to follow the procedures as per the study protocol and agreed to take the investigational product till Day 56, and had the capability of complete compliance and completion of follow-up, were included in the study.

#### Exclusion criteria

Women with severe anemia, previous treatment with hormonal therapy, a history of any bleeding disorders, breast, endometrial, or other gynecological cancer at any time, or any other cancer within the last 5 years were excluded from the study. Participants who had a medical history of smoking, alcoholism, and drug dependence, or hypersensitivity to ARE, were excluded from the study. Participants using vitamin or mineral supplements, nutritional supplements, and or medical foods, estrogen, selective serotonin reuptake inhibitors within 30 days, using prescription medications for acute medical conditions, semi-acute medical conditions, and weight loss were not included in the study. Participants who were pregnant and breastfeeding were excluded. Participants with uncontrolled, unstable comorbidities or taking part in any other clinical trials, or any other condition that the principal investigator believed could compromise the safety of patients, were excluded.

After providing informed consent, 60 women who met the inclusion and exclusion criteria were enrolled in the study.

### 2.4 Sample size calculation

The sample size was calculated based on the primary endpoint of the study. The sample size calculation incorporated testing the hypothesis with a 5% level of significance and adequate power. The assumptions for the parameters, the effect size, and the standard deviation were based on past similar studies. Assuming the mean change in Menopause Rating Scale (MRS) score as 1.67 and 2.1 in the ARE and placebo (PL) arms respectively, the common standard deviation as 0.48, two-sided level of significance as 5%, and power as 90%, the study needed 54 evaluable subjects (27 subjects in each arm) to show superiority of ARE over PL in terms of change in MRS score. However, 60 women (30 in each group) participated in the study.

### 2.5 Randomization & blinding

Block Randomization was carried out using an automated random number generation system (Rando version 1.2 R) with 1:1 allotment. Participants underwent assessments at baseline and day 56. To ensure blinding, ARE and PL capsules were identical in appearance, shape, color, and packaging. The randomization codes were securely concealed in separate envelopes and were only accessed by the investigator after assigning a study number to each participant. The investigator and all personnel involved in data collection and statistical analysis remained blinded to the treatment allocation throughout the study.

### 2.6 Study intervention

Dried roots of *Withania somnifera* (L.) Dunal [Solanaceae; Withaniae radix] was used in this study. Participants received an ARE capsule (a light brown capsule containing 300 mg Ashwagandha root extract powder, Ixoreal Biomed Inc., Los Angeles, USA) (ARE, *n* = 30) or an identical PL capsule (a light brown capsule containing 300 mg starch) (Placebo group, PL, *n* = 30) in a 1:1 ratio as per the randomization schedule. Participants were instructed to take one capsule twice daily after meals (breakfast and dinner) with water for 56 days.

### 2.7 Investigational product details

The investigational product KSM-66 Ashwagandha was obtained from the manufacturer Ixoreal Biomed Inc., Los Angeles, California, USA. KSM-66 is the commercially available highest concentration root-only extract of Ashwagandha, which is produced through a green chemistry (aqueous-extraction process) method that is devoid of any alcohol or chemical solvents. The product is a light yellowish powder and does not have any carcinogenic, teratogenic, or mutagenic effects. The product contains a root-only extract from Ashwagandha, with an optimum amount of withanolides (>5%) precisely estimated by the High-Performance Liquid Chromatography (HPLC) method (Supplementary Figure S1) with an herb to extract ratio of 12:1. The herbs are grown in regions with optimum rainfall (650 mm–750 mm) and proper soil conditions (pH: 7.5–8.0). The PL contained starch powder. Both the products (placebo and the Ashwagandha root extract) were used in the form of an identical gelatin capsule, which was invariable in color, shape, and size. The dosage used for the present study was 300 mg capsules twice daily, both for the Ashwagandha and PL groups. The Ashwagandha root extract used in this study has been classified as Extract Type A in accordance with the Consensus statement on the reporting of pharmacology and physiology studies in natural product research (ConPhyMP) (14). The classification was confirmed using the ConPhyMP interactive tool (https://ga-online.org/best-practice/#conphymp) under the domain of Phytochemical Characterization of Medicinal Plant Extracts.

### 2.8 Study outcomes

Participants were assessed at baseline (visit 1) and day 56 (visit 3) by a trained clinician. At each visit, vital signs were recorded, including systolic and diastolic blood pressure, pulse rate, respiratory rate, and body temperature. Along with these, blood samples were collected to evaluate hematological parameters, liver and kidney functions, and lipid profile.

#### 2.8.1 Primary outcome measures

The MRS is a standardized Health-Related Quality of Life Scale (HRQoL) measure with good psychometric characteristics (15). The MRS and the mean change in the MRS from baseline were descriptively summarized for each visit and treatment.

#### 2.8.2 Secondary outcome measures

##### 2.8.2.1 Change in hormonal parameters and hot flashes

Blood samples were collected at baseline and day 56 to measure serum hormone levels. Hormonal evaluations included serum estradiol, progesterone, LH, and FSH. Samples were drawn into both Ethylenediaminetetraacetic Acid (EDTA) and non-EDTA vials, then centrifuged, and the serum was stored at −80 °C for subsequent analysis. The hormone levels were measured using enzyme-linked immunosorbent assay (ELISA) kits.

Hot flashes were evaluated based on their reported frequency and intensity. Participants rated the severity of each symptom on a scale from 0 (not at all bothersome) to 4 (very bothersome), reflecting their experience over the past month. Hot flashes were determined by comparing scores recorded at baseline and at the end of the study.

##### 2.8.2.2 Change in SF-12 (quality of life)

The 12-Item Short Form Health Survey (SF-12) (16) is used to measure quality of life related to health. This survey produced two main scores: the Physical Component Summary (PCS) and the Mental Component Summary (MCS). A known scoring systems was used to figure out these scores. A score of 50 or more means that health is better than average. A score of 40–49 means that health is slightly worse than average. A score of less than 40 means that health is really bad, either physically or mentally. Scores below 30 show that health-related quality of life is very bad. These interpretations were used to look at and compare the physical and mental health of all the people in the study.

##### 2.8.2.3 Patient global assessment of tolerability to therapy (PGATT)

It was assessed based on a four-point rating scale as follows: 1 = excellent tolerability, 2 = good tolerability, 3 = average tolerability, and 4 = poor tolerability. This scoring was done by the patients. The adherence to the assigned regimen was assessed and verified by study personnel through recording the dosing of the investigational products (17).

##### 2.8.2.4 Perceived stress scale (PSS-10)

The Perceived Stress Scale (17) score is a 10-item questionnaire designed to evaluate the degree to which individuals perceive their lives as stressful over the past month. Each item is rated on a five-point Likert scale ranging from “never” to “very often”.

#### 2.8.3 Safety assessment

Adverse events observed by the investigator or reported by the participants were documented for each participant throughout the study as part of a safety evaluation. Serum biochemical parameters were assessed at baseline and day 56 for any effects on kidney (serum creatinine, blood urea nitrogen) and the liver (serum alanine transaminase [ALT], aspartate transaminase [AST], alkaline phosphatase [ALP], bilirubin). Hematological assessments included haemoglobin, Red Blood Cell count, haematocrit, total leukocyte count, lymphocytes, monocytes, eosinophils, basophils, absolute neutrophils, and platelet count.

### 2.9 Statistical methods

All relevant statistical calculations were carried out with Stata 13.0 IC (Stata Corp. USA). Since all women completed the study as per the study protocol, efficacy and safety analysis were performed on all patient's dataset (*n* = 60). The analyses used two-sided tests and a *p*-value of less than 0.05 was considered statistically significant. For categorical variables, the number and percentage of subjects within each category (with category for missing data as needed) were provided and *p*-values were calculated and compared using the Chi-square test between the groups (ARE vs. PL). For continuous variables, the number of subjects, mean, median, standard deviation (SD), minimum and maximum values were provided. Paired *t*-test was used for the within group (Baseline-Day 56) comparison, Two-independent samples *t*-test was used for the between group (ARE vs. PL) comparison at Baseline and Day 56 and Analysis of Covariance (ANCOVA) was used for between-group comparisons (ARE vs. PL) after controlling for baseline.

## 3 Results

A total of 73 participants were screened for eligibility for enrollment. Of these, 60 participants met the inclusion criteria and were randomized in a 1:1 ratio to receive either ARE (*n* = 30) or PL (*n* = 30).

**Figure 1:**
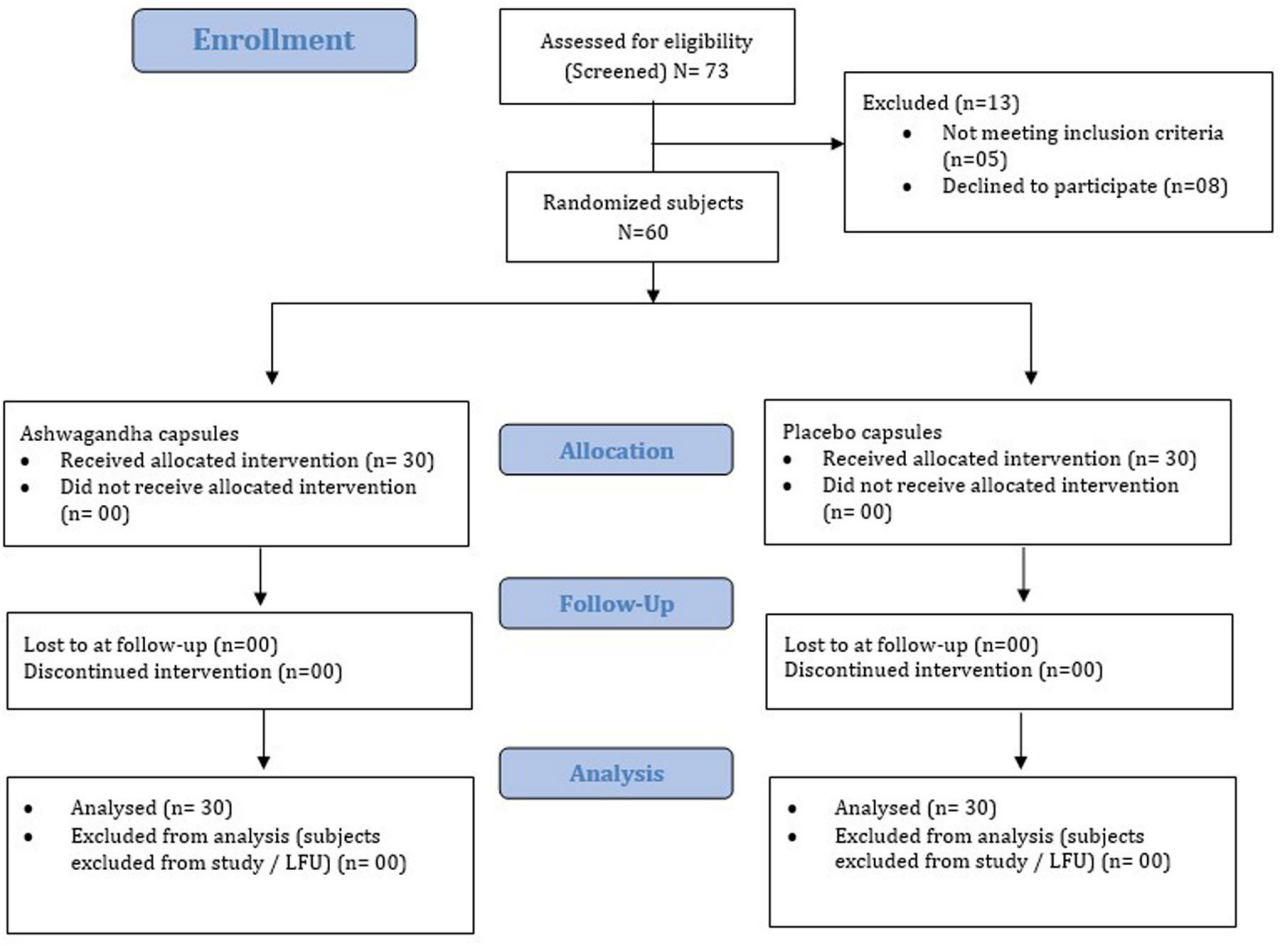
Consort flow representation of the patient enrollment, allocation, follow-up and analysis. LFU, lost to follow-up.

During the study period, no participants from either group were withdrawn due to follow-up loss or failure of medication adherence (Figure 1). Thus, for efficacy and safety data analysis, the Per protocol (PP) population and Intent to treat (ITT) population are ARE (*n* = 30) and PL (*n* = 30), respectively. A consort flow diagram illustrating the participant disposition is presented in Figure 1.

### 3.1 Demographics and baseline data (ITT dataset)

Table 1 presents the baseline demographic and medical history profile for randomized participants (*n* = 60) included in the ITT analysis. No statistically significant difference between ARE and PL groups was detected, for any measurable parameters at baseline. The mean age for ARE was 49.8 ± 2.4 years and for PL was 50.0 ± 2.3 years (*p* = 0.700), indicating no significant difference. The mean BMI for ARE was 24.9 ± 2.4 and for PL was 25.0 ± 2.5 (*p* = 0.955), also showing no significant difference. No statistically significant difference between ARE and PL groups was detected in the prevalence of co-morbidities such as anxiety (ARE: 6.7%, PL: 3.3%, *p* = 0.549), constipation (ARE: 3.3%, PL: 0%, *p* = 0.319), diabetes (ARE: 6.7%, PL: 3.3%, *p* = 0.549), hypertension (ARE: 26.7%, PL: 16.7%, *p* = 0.351), and obesity (ARE: 50%, PL: 43.3%, *p* = 0.605).

**Table 1:** Demographic and medical history characteristics of participants at baseline.

Characteristic	ARE group (*n* = 30)	PL group (*n* = 30)	*p* Value
Demographics			
Age (years)	49.8 ± 2.4	50.0 ± 2.3	0.700
BMI (kg/m^2^)	24.9 ± 2.4	25.0 ± 2.5	0.955
Medical History			
Anxiety, *n* (%)	2 (6.7%)	1 (3.3%)	0.549
Constipation, *n* (%)	1 (3.3%)	0 (0%)	0.319
Diabetes, *n* (%)	2 (6.7%)	1 (3.3%)	0.549
Hypertension, *n* (%)	8 (26.7%)	5 (16.7%)	0.351
Obesity, *n* (%)	15 (50.0%)	13 (43.3%)	0.605

Values are presented as mea*n* ± standard deviation or number (%). ARE, Ashwagandha Root Extract; PL, Placebo Group.

### 3.2 Vital parameters

Table 2 shows the vital parameters at baseline (day 1) and end of study period (day 56) with no significant difference between the two groups (*p* > 0.05). The physical examination of patients in both groups was found to be within normal limits.

**Table 2:** Vital parameters and laboratory values at baseline and end of study.

Vital parameters and laboratory values	ARE (*n* = 30)			PL (*n* = 30)		
	Day 1	Day 56	*p*	Day 1	Day 56	*p*
	Mean (SD)	Mean (SD)		Mean (SD)	Mean (SD)	
SBP (mmHg)	142.50 (20.5)	140.8 (19.6)	0.748	129.8 (16.8)	130.0 (16.4)	0.969
DBP (mmHg)	90.0 (10.7)	88.8 (9.3)	0.653	84.5 (7.8)	84.3 (7.2)	0.932
Pulse Rate (/min)	95.3 (15.4)	94.1 (15.4)	0.770	84.7 (7.0)	84.9 (7.0)	0.927
Respiration Rate (/min)	20.5 (2.8)	20.4 (2.9)	0.858	19.4 (2.7)	19.4 (2.7)	>0.999
Temperature (˚C)	36.5 (0.4)	36.5 (0.4)	>0.999	36.6 (0.4)	36.6 (0.4)	0.812
Haemoglobin (g/dL)	12.2 (0.8)	12.6 (1.0)	0.177	11.7 (0.9)	11.7 (0.9)	0.753
RBC count (x10^6/µL)	4.7 (0.5)	4.8 (0.5)	0.336	4.7 (0.6)	4.6 (0.5)	0.823
Haematocrit (PCV) (%)	42.9 (3.5)	43.2 (3.2)	0.79	43.0 (3.4)	41.1 (4.7)	0.084
TLC (cells/µL)	4,740.0 (447.7)	4,736.7 (426.3)	0.997	4,846.7 (337.1)	4,693.3 (865.4)	0.37
Lymphocytes (%)	29.2 (5.7)	29.1 (5.4)	0.926	32.3 (6.6)	32.9 (6.4)	0.752
Monocytes (%)	4.7 (1.7)	4.7 (1.7)	0.958	5.5 (1.3)	5.4 (1.3)	0.775
Eosinophils (%)	3.3 (1.3)	3.4 (1.2)	0.757	3.1 (1.0)	3.2 (1.0)	0.518
Basophils (%)	0.5 (0.4)	0.5 (0.3)	0.848	0.5 (0.2)	0.5 (0.2)	0.533
Absolute Neutrophils (%)	3,930.0 (490.0)	3,933.3 (488.0)	0.979	3,916.7 (472.2)	3,893.3 (441.1)	0.844
Platelet Count (cells/µL)	1,59,833.3 (10,627.2)	1,60,350.0 (9,150.2)	0.841	1,59,566.7 (12,475.3)	1,60,100.0 (0.5)	0.861
Total bilirubin (mg/dL)	0.5 (0.3)	0.5 (0.3)	0.897	0.6 (0.2)	0.6 (0.2)	0.46
AST/SGOT (U/L)	12.2 (3.9)	11.7 (3.5)	0.553	12.3 (3.4)	13.0 (3.2)	0.44
ALT/SGPT (U/L)	10.3 (3.5)	10 (3.2)	0.7	12.4 (3.2)	13.1 (3.4)	0.393
ALP (U/L)	101.4 (13.6)	103.9 (15.5)	0.51	101.5 (17.3)	103.5 (20.1)	0.681
BUN (mg/dL)	9.5 (2.0)	9.5 (2.0)	>0.999	9.4 (1.5)	9.4 (1.5)	>0.999
Creatinine (mg/dL)	0.8 (0.2)	0.8 (0.2)	0.803	0.7 (0.2)	0.7 (0.2)	0.702
Total Cholesterol (mg/dL)	192.0 (30.3)	192.0 (30.3)	>0.999	190.8 (16.2)	191.8 (16.3)	0.824

ARE, Ashwagandha Root Extract; PL, Placebo; SD, Standard Deviation; PCV, Packed Cell Volume; TLC, Total Leukocyte Count; AST/SGOP, Aspartate aminotransferase; ALT/SGPT: Alanine aminotransferase; ALP, Alkaline phosphatase; BUN, Blood Urea Nitrogen.

### 3.3 Efficacy assessment

#### 3.3.1 Menopause rating scale

**Figure 2:**
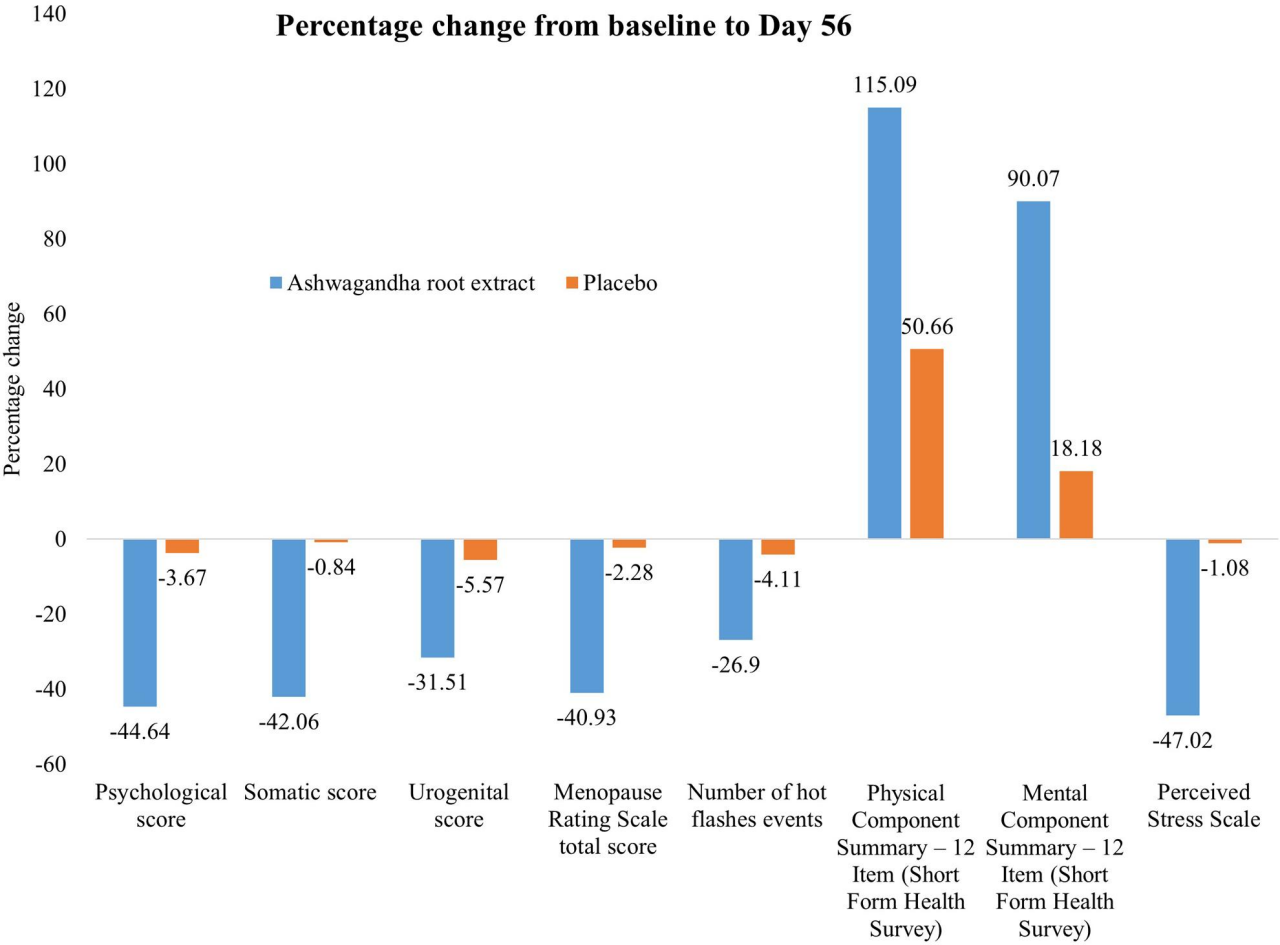
Effect of Ashwagandha on menopausal symptoms and quality of life over 56 days.

Table 3 presents the MRS and its sub-domain scores at baseline (day 1) and end of study (day 56). A significant reduction in all sub-domain scores, such as psychological, somatic, and urogenital scores, was seen in the ARE group as compared to the PL group (*p* < 0.0001). There was a significant reduction in mean (SD) of the total MRS scores with ARE from 31.37 (1.45) at baseline to 18.53 (2.29) at day 56, compared to PL from 30.73 (2.50) to 30.03 (2.55), with a *p* value of <0.0001. The effect sizes (Cohen's *d*) observed across all domains of the MRS indicate a consistently strong and clinically meaningful impact of the intervention in the ARE group compared to the PL group over 56 days. Figure 2 presents the percentage change from baseline to Day 56 for the individual domain and the total score.

**Table 3:** Menopause rating scale scores.

MRS and sub-domain scores	ARE (*N* = 30)	PL (*N* = 30)	Difference	Effect size Cohen’s “*d*”	*p*-Value
	Mean (SD)	Mean (SD)			
Psychological score				=d/SD	
Baseline	12.77 (1.41)	12.58 (1.42)	0.19	0.13	0.605
Day 56	7.07 (1.05)	12.33 (1.52)	−5.26	−5.01	<0.0001*
Change from baseline	−5.70 (1.97)	−0.25 (0.55)	−5.45	−2.76	<0.0001*
Somatic score					
Baseline	12.03 (1.25)	11.87 (1.45)	0.16	0.13	0.6488
Day 56	6.97 (1.07)	11.77 (0.90)	−4.80	−4.49	<0.0001*
Change from baseline	−5.06 (0.99)	−0.10 (3.01)	−4.96	−1.65	<0.0001*
Urogenital score					
Baseline	6.57 (0.82)	6.28 (1.13)	0.29	0.35	0.2599
Day 56	4.50 (1.01)	5.93 (1.26)	−1.43	−1.42	<0.0001*
Change from baseline	−2.07 (1.04)	−0.35 (0.71)	−1.72	−1.65	<0.0001*
MRS total score					
Baseline	31.37 (1.45)	30.73 (2.50)	0.64	0.44	0.235
Day 56	18.53 (2.29)	30.03 (2.55)	−11.50	−5.02	<0.0001*
Change from baseline	−12.84 (4.60)	−0.35 (0.71)	−12.14	−2.64	<0.0001*

ARE, Ashwagandha Root Extract; PL, Placebo; SD, Standard Deviation; MRS, Menstrual Rating Scale.

* Statistically significant at <0.0001.

#### 3.3.2 Hormone levels and hot flashes

Tables 4 presents serum hormone levels and the occurrence of hot flashes. There were statistically significant changes observed in all parameters between the two groups (*p* < 0.001). Serum estradiol and serum progesterone levels increased in the ARE group, while luteinizing hormone (LH) and FSH increased in the PL group. In both groups, there was no significant decrease in hot flashes score (number of events) at day 56 as compared to baseline (*p* > 0.05). Nevertheless, this reduction was statistically significant when compared between groups (*p* < 0.001). The effect size analysis across hormonal biomarkers, and hot flash frequency indicates a strong and consistent treatment effect in the ARE group compared to the PL group over 56 days. Figure 2 presents the percentage change from baseline to Day 56 for hot flashes.

**Table 4:** Hormone levels and scores for hot flashes.

Hormones (units)		ARE (*N* = 30)	PL (*N* = 30)	Difference	Effect size Cohen's “*d*”	“*p*” value between group
		Mean (SD)	Mean (SD)			
Serum estradiol (pg/mL)	Baseline—*Mean (SD)*	22.20 (3.80)	22.20 (3.80)	0.00	0.00	>0.999
	Day 56—*Mean (SD)*	23.02 (3.50)	21.6 0 (3.30)	1.60	0.46	0.068
	Change—*Mean change* ± *S*D *(*“*p*” *value Within group)*	1.00 ± 6.00 (<0.001)*	−0.60 ± 6.00 (0.012)*	1.60	0.27	<0.001*
Serum progesterone (ng/mL)	Baseline—*Mean (SD)*	0.28 (0.08)	0.30 (0.07)	−0.02	−0.30	0.229
	Day 56—*Mean (SD)*	0.43 (0.19)	0.30 (0.08)	0.13	0.70	0.001*
	Change—*Mean change* ± *SD (“p” value Within group)*	0.15 ± 0.96 (<0.001)*	−0.01 ± 0.39 (0.601)	0.16	0.16	<0.001*
Serum LH (mIU/mL)	Baseline—*Mean (SD)*	36.80 (8.60)	34.80 (7.70)	2.00	0.23	0.328
	Day 56—*Mean (SD)*	35.50 (8.60)	35.60 (7.70)	−0.10	−0.01	0.948
	Change—*Mean change* ± *S*D *(“p” value Within group)*	−1.30 ± 0.60 (<0.0001)*	0.80 ± 0.60 (0.001)*	−2.10	−0.35	<0.001*
Serum FSH (mIU/mL)	Baseline—*Mean (SD)*	61.90 (16.10)	58.20 (15.30)	3.70	0.23	0.362
	Day 56—*Mean (SD)*	60.90 (15.90)	58.60 (15.40)	2.30	0.14	0.574
	Change—*Mean change* ± *SD (“p” value Within group)*	−1.00 ± 0.60 (<0.001)*	0.40 ± 6.00 (0.034)*	−1.40	−0.23	<0.001*
No. of hot flashes events	Baseline—*Mean (SD)*	14.50 (2.00)	14.60 (2.20)	−0.10	−0.05	0.855
	Day 56—*Mean (SD)*	10.60 (2.4)	14.00 (2.30)	−3.40	−1.42	0.078
	Change—*Mean change* ± *SD (“p” value Within group)*	−3.90 ± 15.00 (<0.001)*	−0.60 ± 6.00 (0.001)*	−3.30	−0.22	<0.001*

ARE, Ashwagandha Root Extract; PL, Placebo; SD, Standard Deviation; FSH, Follicle stimulating hormone; LH, Luteinizing hormone; IU, International units; dL, Deciliter.

* Statistically significant.

#### 3.3.3 Short form survey (SF-12)

Table 5 presents the SF-12 (PCS-12, MCS-12) scores at baseline (day 1) and the end of study period (day 56). There was marked improvement in all SF-12 scores in both groups and between the groups (*p* < 0.001). Assessment of quality of life (SF-12) over 56 days revealed significant and clinically meaningful improvements in the ARE group compared to PL, as reflected in large between-group effect sizes and statistically robust differences. Figure 2 presents the percentage change from baseline to Day 56.

**Table 5:** SF-12 (PCS-12, MCS-12) and PSS scores.

Parameters (scales)		ARE (*N* = 30)	PL (*N* = 30)	Difference	Effect size Cohen’s “d”	*“p” value between group*
		Mean (SD)	Mean (SD)			
PCS-12 (SF-12)	Baseline—*Mean (SD)*	23.20 (1.50)	22.70 (1.10)	0.50	0.33	0.193
	Day 56—*Mean (SD)*	49.90 (2.00)	34.20 (3.90)	15.70	7.85	<0.001*
	Change—*Mean change* ± *S*D *(“p” value Within group)*	26.7 ± 15.00 (<0.001*)	11.5 ± 21.00 (<0.001*)	15.20	0.72	<0.001*
MCS-12 (SF-12)	Baseline—*Mean (SD)*	28.20 (7.9)	30.80 (6.60)	-2.60	-0.33	0.175
	Day 56—*Mean (SD)*	53.60 (2.5)	36.40 (6.50)	17.20	6.88	<0.001*
	Change—*Mean change* ± *S*D *(“p” value Within group)*	25.50 ± 45.00 (<0.001*)	5.60 ± 36.00 (<0.001*)	19.90	0.44	<0.001*
PSS	Baseline—*Mean (SD)*	28.50 (3.10)	27.70 (3.10)	0.80	0.26	0.281
	Day 56—*Mean (SD)*	15.10 (3.00)	27.40 (3.00)	-12.30	-4.10	<0.001*
	Change—*Mean change* ± *SD (“p” value Within group)*	-13.40 ± 21.00 (<0.001*)	-0.20 ± 6.00 (0.326)	-13.20	-0.63	<0.001*

ARE, Ashwagandha Root Extract; PL, Placebo; SD, Standard Deviation; PCS-12; Physical component score; MCS-12; Mental component score; PSS, Perceived stress scale.

* Statistically significant.

#### 3.3.4 Patients global assessment of tolerability to therapy (PGATT)

**Figure 3:**
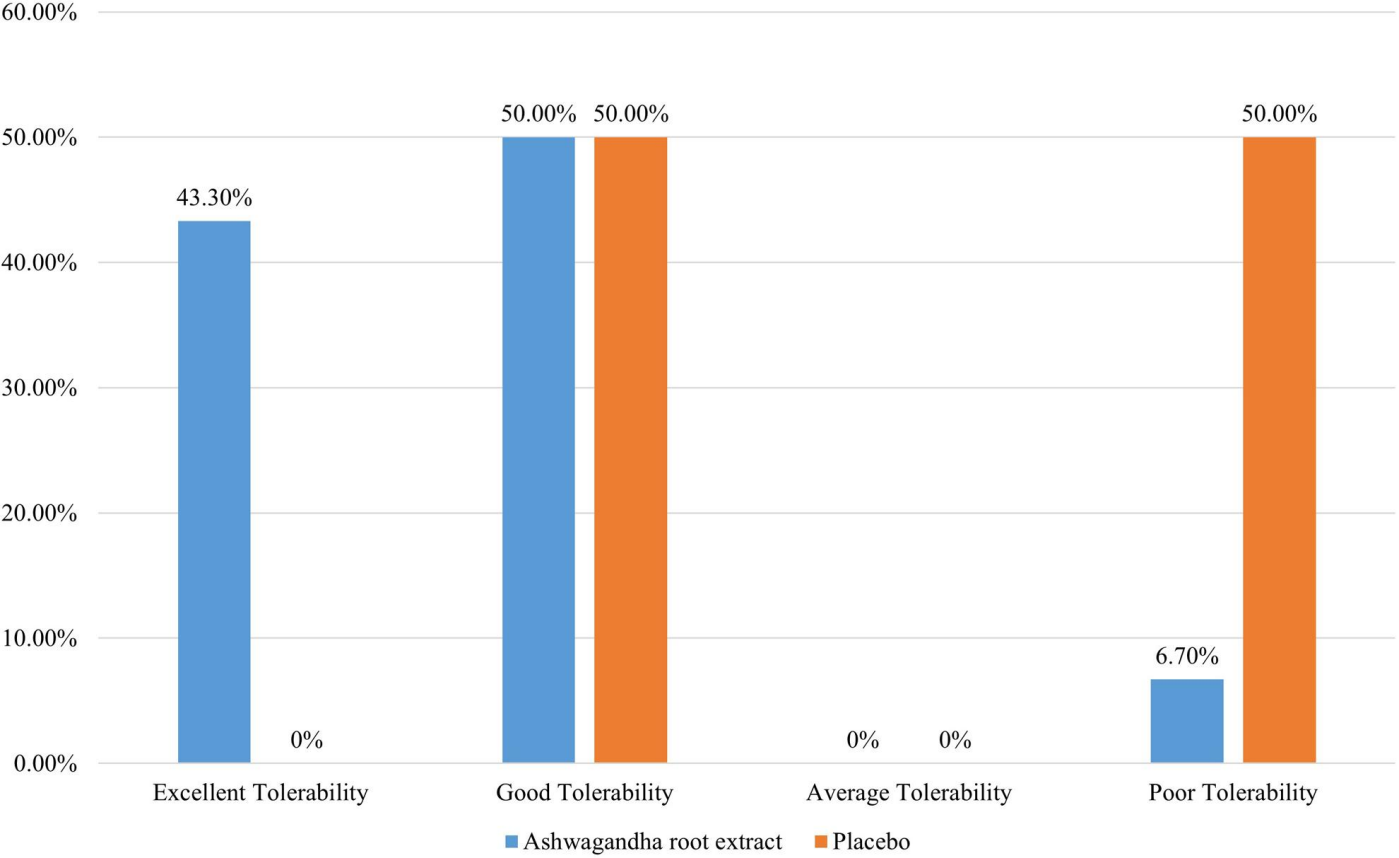
Patients global assessment of tolerability to therapy.

Table 6 presents the Patient's Global Assessment of Tolerability to Therapy (PGATT) parameters. Most of the patients in the ARE group reported good to excellent tolerance for ARE compared to PL (*p* < 0.001). A total of twenty-eight patients (93.3%) in the ARE group rated the tolerability of ARE as “good” to “excellent”. Poor tolerability was reported in 15 participants (50.0%) in the PL group. Figure 3 presents the percentage tolerability of ARE in comparison to PL.

**Table 6:** PGATT parameters at baseline and end of study period.

PGATT parameters	ARE (*N* = 30)	PL (*N* = 30)	Chi-square Test	
	*No. (%)*	*No. (%)*	*χ2*	*“p”*
Excellent Tolerability	13 (43.3%)	0 (-)	22.941	<0.001*
Good Tolerability	15 (50.0%)	15 (50.0%)		
Average Tolerability	0 (-)	0 (-)		
Poor Tolerability	2 (6.7%)	15 (50.0%)		

ARE, Ashwagandha Root Extract; PL, Placebo.

* Statistically significant.

#### 3.3.5 Perceived stress scale (PSS-10)

Table 5 shows the PSS-10 scores at baseline (day 1) and end of study (day 56). There was a significant improvement in all PSS-10 score in the ARE group and changes were statistically significant between the groups (*p* < 0.001). There was reduction (improvement) in mean (SD) PSS-10 score from baseline to day 56 in ARE group (*p* < 0.001) with a negligible, statistically non-significant, change from baseline to day 56. Assessment of perceived stress using the PSS over 56 days showed a significant and clinically relevant reduction in the ARE group compared to the PL group. Figure 2 presents the percentage change from baseline to Day 56.

### 3.4 Safety assessment and treatment compliance

A total of 3 women (1 in the ARE group and 2 in the PL group) reported adverse events. One woman in the ARE group reported a cough and cold, while two women in the PL group reported stomachache and indigestion, respectively. All events were of mild severity, were not associated with the study treatments, and completely resolved with or without symptomatic treatment. None of the women discontinued treatment due to adverse events, and treatment compliance was 100% in both groups.

## 4 Discussion

Menopause typically occurs between the ages of 45–52 years and is identified by changes in hormones that lead to the stoppage of menstrual cycles (18, 19). By 2030, almost 1.2 billion women globally will be menopausal or postmenopausal, with 47 million new entrants annually. Menopause lasts for one-third of a woman's life (20, 21). Vasomotor symptoms, experienced by over 80% of women, can last from 5 to 15 years, significantly impacting sleep, mood, cognition, and quality of life (22–24). MHT has historically been the primary treatment, while beneficial effects on vasomotor dysfunction have been reported with estrogen, gabapentin, paroxetine and clonidine, but health concerns lead many women to seek alternative choices (25).

A study conducted by Gopal et al. demonstrated the effects of 300 mg of Ashwagandha root extract twice daily on climacteric symptoms in perimenopausal women for eight weeks (10). Of these 91 participants, 46 women received Ashwagandha and showed significant improvements in symptoms within four weeks, including hot flashes. At 8 weeks, the Ashwagandha group showed a decrease in overall MRS scores, and a statistically significant increase in serum estradiol and a decrease in serum FSH and LH levels compared to the PL group (11). A study by Modi et al., evaluated *Ashokarishta*, *Ashwagandha Churna*, and *Praval Pishti* in managing menopausal symptoms, with the result showing reductions in MRS and Menopause Specific Quality of Life (MENQoL) questionnaire scores in 51 women (26). One of the study reported better results with Shatavari when compared to Ashwagandha (27). The current study aligns with similar findings, where a statistically significant reduction in mean MRS score from 31.37 to 18.53 was noted (*p* < 0.001). This was associated with a statistically significant increase in serum estradiol (*p* < 0.001) and progesterone (*p* < 0.001), and a significant reduction in serum FSH (*p* < 0.001) and LH (*p* < 0.001) concentrations, as compared with the PL. The statistically significant hormonal changes observed in this study may result from both a direct endocrine effect and an indirect effect mediated by a reduction in physiological stress (23, 24).

There was also a statistically significant reduction in hot flash events (*p* < 0.05) in the ARE group, as compared to the PL group. While previous studies used MENQoL for quality-of-life assessment, the present study used SF-12 and PSS-10 scales to focus on physical, mental, and psychological well-being. The present study results showed improvement in the quality of life of menopausal women in the ARE group, reflecting statistically significant improvements in PCS-12, MCS-12, and PSS-10 scores (*p* < 0.001).

ARE is reported to be safe for human use with a daily dosage of up to 1,000 mg (27). The present data support this finding, as no serious adverse events related to ARE were noted and more than 90% of the participants reported tolerability of ARE as good to excellent. Further, treatment compliance was 100% in the present study. These findings add crucial evidence to the ARE safety and efficacy assessment in addressing menopause symptoms.

The observed benefits of ARE in menopausal women may be attributed to its multifaceted mechanisms of action. Ashwagandha is known for its adaptogenic properties, which help the body cope with physical and psychological stress, potentially through modulation of the hypothalamic-pituitary-adrenal axis (28). Additionally, its GABA-mimetic activity may contribute to improvements in sleep, mood, and anxiety, while anti-inflammatory and antioxidant pathways could alleviate systemic stress-related symptoms (7–9).

The strength of the present study lies in its robust randomized, double-blind, and placebo-controlled design, which enhances the reliability of the findings. The intervention used in this study was a branded ARE (KSM-66®). Therefore, the results may not be directly extrapolated to other ARE preparations that have different phytochemical profiles or standardization methods.

The limitations of the study include the short duration, small sample size, and limited ability to detect smaller effects. The cohort was homogeneous and well-defined, drawn from a specific cross-section of society, which, along with the study setting, may restrict the generalizability of the findings. Finally, blinding effectiveness was not formally assessed, although the intervention was low-risk and the outcomes were largely objective. While ARE demonstrated beneficial effects on stress and well-being in this study, it is important to consider the potential contribution of PL effects, which are commonly observed in interventions involving subjective outcomes such as perceived stress and quality of life. Future research conducted in a real-world setting with participants representing a wider range of demographics, occupations, and socio-economic statuses is necessary to validate and extend the applicability of the findings. In addition, the study's eight-week duration may limit the ability to conclude long-term benefits, warranting consideration in future investigations.

## 5 Conclusions

Ashwagandha (*Withania somnifera*) appears to be a promising herbal intervention for the management of menopausal symptoms. The present study demonstrated statistically significant improvement in the MRS scale, selected hormonal parameters, hot flash, SF-12 scale, and perceived stress, over a duration of 56 days without adverse effects. While these findings support the therapeutic potential of ARE, further well-powered, long-term clinical trials are warranted to confirm efficacy, elucidate the mechanism of action, and establish its use as an effective and safe alternative to hormone-based therapies.

## Data Availability

The raw data supporting the conclusions of this article will be made available by the authors, without undue reservation.
