# Supplementary figures and images for: A prospective, randomized, double-blind, placebo-controlled study on efficacy and safety of Ashwagandha root extract (Withania somnifera) for managing menopausal symptoms in women
Source: Front Reprod Health. 2026 Jan 5;7:1647721. doi: 10.3389/frph.2025.1647721 (PMC12812913; doi:10.3389/frph.2025.1647721)

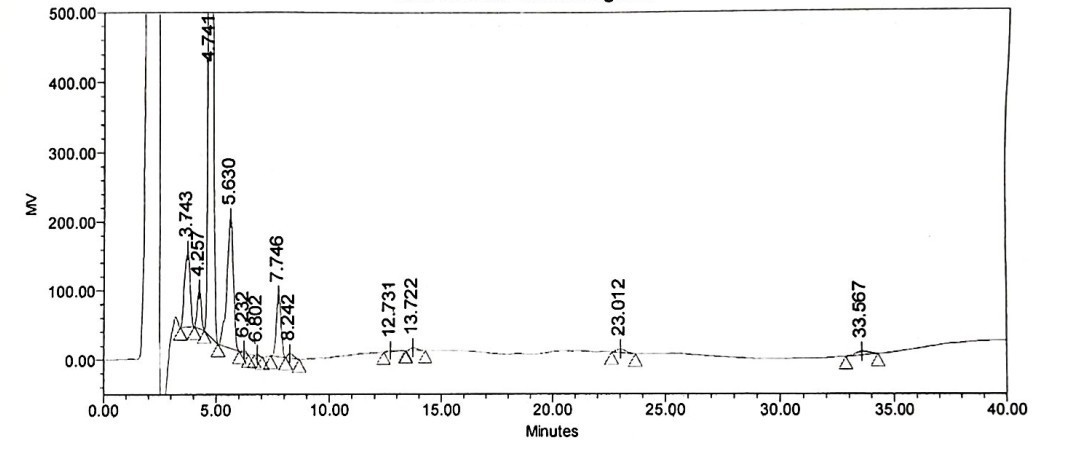

Supplement: Supplementary file 3 [file Image1.jpeg]
